# Infarct-preconditioning exosomes of umbilical cord mesenchymal stem cells promoted vascular remodeling and neurological recovery after stroke in rats

**DOI:** 10.1186/s13287-022-03083-9

**Published:** 2022-07-28

**Authors:** Yi-Chao Ye, Zhe-Han Chang, Peng Wang, You-Wei Wang, Jun Liang, Chong Chen, Jing-Jing Wang, Hong-Tao Sun, Yi Wang, Xiao-Hong Li

**Affiliations:** 1 Academy of Medical Engineering and Translational Medicine, Tianjin University, Tianjin, 300072 China; 2 Tianjin Key Laboratory of Neurotrauma Repair, Characteristic Medical Center of People’s Armed Police Forces, Tianjin, 300162 China; 3 Health Management Department, Tianjin Hospital, Tianjin University, Tianjin, 300299 China; 4 Neurology Department, Tianjin Hospital, Tianjin University, Tianjin, 300299 China

**Keywords:** Preconditioning, Brain extract, Exosomes, Stroke, Umbilical cord mesenchymal stem cells, Vascular remodeling

## Abstract

**Background:**

Stroke is the leading cause of disability worldwide, resulting in severe damage to the central nervous system and disrupting neurological functions. There is no effective therapy for promoting neurological recovery. Growing evidence suggests that the composition of exosomes from different microenvironments may benefit stroke. Therefore, it is reasonable to assume that exosomes secreted in response to infarction microenvironment could have further therapeutic effects.

**Methods:**

In our study, cerebral infarct tissue extracts were used to pretreat umbilical cord mesenchymal stem cells (UCMSC). Infarct-preconditioned exosomes were injected into rats via tail vein after middle cerebral artery occlusion (MCAO). The effect of infarct-preconditioned exosomes on the neurological recovery of rats was examined using Tunel assay, 2,3,5-triphenyltetrazolium chloride (TTC) assay, magnetic resonance imaging (MRI) analyses, modified Neurological Severity Score (mNSS), Morris water maze (MWM), and vascular remodeling analysis. Mi-RNA sequencing and functional enrichment analysis were used to validate the signal pathway involved in the effect of infarct-preconditioned exosomes. Human umbilical vein endothelial cells (HUVECs) were co-cultured with the isolated exosomes. Cell Counting Kit-8 (CCK-8) assay, scratch healing, and Western blot analysis were used to detect the biological behavior of HUVECs.

**Results:**

The results showed that compared with normal exosomes, infarct-preconditioned exosomes further promoted vascular remodeling and recovery of neurological function after stroke. The function of upregulated miRNAs and their target genes which is beneficial to vascular smooth muscle cells verified the importance of vascular remodeling in improving stroke. Better resistance to oxygen–glucose deprivation/reoxygenation (OGD/R), reduced apoptosis, and enhanced migration were observed in infarct-preconditioned exosomes-treated umbilical vein endothelial cells.

**Conclusions:**

Our results demonstrated that infarct-preconditioned exosomes promoted neurological recovery after stroke by enhancing vascular endothelial remodeling, suggested that infarct-preconditioned exosomes could be a novel way to alleviate brain damage following a stroke.

**Supplementary Information:**

The online version contains supplementary material available at 10.1186/s13287-022-03083-9.

## Introduction

Stroke causes localized blood supply disorders, resulting in ischemia, hypoxia, edema and necrosis, and varying degrees of neurological impairment. It is also a global disease with high morbidity, mortality, and healthcare costs [1]. Although many neuroprotective agents have been studied, they still not succeed [2]. Recently, a large number of studies have focused on vascular changes after ischemic stroke, as recovery of function is highly dependent on an effective blood supply. Vascular remodeling is essential to maintain physical function and positively correlated with survival after stroke [3–7]. A study demonstrated that interleukin-6 (IL-6)-preconditioning-neural stem cells significantly reduced infarcts and promote angiogenesis [8]. Umbilical cord mesenchymal stem cells (UCMSC) are widely used for functional cell replacement after stroke because of its immunomodulatory and self-renewal capacity [9–12]. However, their efficacy may be limited due to the death of transplanted cells and the inflammatory response of the surrounding tissue. Recent studies have shown that transplanted MSCs exert therapeutic effects through paracrine mechanisms and exosomes play an important role in this process [13]. Exosomes are bilayer vesicles between 50 and 120 nm in diameter that can be secreted by different types of cells. It has been reported that exosomes can achieve therapeutic effects similar to the cells where they are derived from [14–16]. It has been shown that MSC-derived exosomes promote functional recovery, angiogenesis, neuroregeneration, and neurovascular plasticity after stroke [17–20].

Exosomes obtained from MSCs preconditioned with different microenvironments contain different active substances [18, 21]. After stroke, exosomes were involved in different responses in the infarct area by crossing the blood–brain barrier (BBB) [22–24]. Exosomes after stroke have important functions in neuroprotection, angiogenesis, and neurogenesis [25]. Exosomes from hypoxic-preconditioning MSCs contained more vascular endothelial growth factor (VEGF) and significantly enhanced cognitive recovery after traumatic brain injury (TBI) in rats [19]. Recent study has confirmed that exosomal microRNA-126 from remote ischemic-preconditioning (RIPC) serum is involved in hypoxia tolerance in SH-SY5Y cells by downregulating DNA methyltransferase 3 beta (DNMT3B) [26]. A previous study confirmed that MALAT1 Up-regulator polydatin protects brain microvascular integrity and ameliorates stroke through C/EBPβ/MALAT1/CREB/PGC-1α/PPARγ pathway. Similar to this study, our currently results demonstrated that serum exosomes from hypoxic-preconditioning mice reduced autophagy-related cognitive dysfunction by enhancing the expression of lncRNA metastasis-associated lung adenocarcinoma transcript 1 (MALAT1) [27]. Exosomes from remote ischemic postconditioning human umbilical vein endothelial cells (HUVECs) protected neurons against hypoxia/reoxygenation-induced injuries by suppressing miR-21-3p [28]. In a study, hypoxic postconditioning was found to protect cardiomyocytes against oxygen–glucose deprivation/reoxygenation (OGD/R)-induced injury by regulating the level of lncRNA H19 [29]. Thus, exosomes secreted by MSCs in response to microenvironment may be essential for the treatment of cerebral infarction and improvement in neurological function.

In order to mimic the complex microenvironment during cerebral infarction, the brain tissue extracted from cerebral infarction was used to preconditioning UCMSCs. We explored the effects of infarct-preconditioned exosomes on recovery after stroke in vitro and in vivo. We found that infarct-preconditioned exosomes exhibited powerful neural repairing capability via mediating vascular remodeling after stroke. The enrichment of miRNA functional pathways associated with vascular neogenesis and the reduction in apoptosis in HUVECs after OGD/R further emphasized the importance of vascular remodeling in neurological repair. Infarct-preconditioned exosomes were proved to be an effective composition to promote functional recovery in central nervous system after stroke.

## Materials and methods

### Animals

8-week-old Sprague–Dawley rats (male, 250 ± 30 g) were purchased from SPF Biotechnology Co. Ltd. (RRID: RGD_737805, Beijing, China). All rats received humane care. The protocol was approved by the Research Animal Ethics Committee of People’s armed police (approval code 23658/42). All rats were kept in a 12-h light/dark cycle with free access to food and water throughout the study (22 ± 2 °C, 50% humidity). The present study was not pre-registered. Randomization was performed with the online tool QuickCalcs from GraphPad. Rats were coded and assigned randomly to different groups for simple randomization by using: "Random numbers" and "Randomly assign subjects to groups," in QuickCalcs. The grouping of rats, methods, and the number used in each group are shown in Table 1. No sample calculation was performed; the sample size was verified for sufficient power by post hoc power analysis. This study was exploratory. The values above or below the mean ± 2σ were excluded from the data; 6 rats were excluded and 7 died during experiments. Timeline of experimental procedure is shown in Additional file 1: Fig. S1.

**Table 1:** Group allocation and animal numbers

Technique for study	Sham	MCAO	EXO	N-EXO	I-EXO
EXO injection	–	–	*n* = 35	*n* = 35	*n* = 35
Died	–	*n* = 2	*n* = 2	*n* = 1	*n* = 2
Excluded	–	*n* = 1	*n* = 1	*n* = 2	*n* = 2
TTC	*n* = 3	*n* = 3	*n* = 3	*n* = 3	*n* = 3
MRI	*n* = 3	*n* = 3	*n* = 3	*n* = 3	*n* = 3
Tunel	*n* = 3	*n* = 3	*n* = 3	*n* = 3	*n* = 3
mNSS	*n* = 9	*n* = 9	*n* = 9	*n* = 9	*n* = 9
MWM	*n* = 10	*n* = 10	*n* = 10	*n* = 10	*n* = 10
IF	*n* = 3	*n* = 3	*n* = 3	*n* = 3	*n* = 3

*MCAO* middle cerebral artery occlusion, *EXO* normal exosome, *N-EXO* exosomes from UCMSCs which are preconditioned by normal cerebral tissue extracts, *I-EXO* exosomes from UCMSCs which are preconditioned by cerebral infarcted tissue extracts, *TTC* 2,3,5-triphenyl tetrazolium chloride, *MRI* cranial magnetic resonance imaging, *mNSS* modified Neurological Severity Scores, *MWM* Morris water maze, *IF* immunofluorescence

### MCAO model and cerebral tissue extracts

Middle cerebral artery occlusion (MCAO) was induced in rats according to the previous descriptions [30]. In brief, rats were anesthetized with isoflurane inhalation (3% for induction, 1.5% for maintenance (v/v)) to ensure sufficient anesthesia during the whole procedure. The right common carotid artery (CCA) and internal carotid artery (ICA) were fully exposed. A 6-cm surgical thread was inserted to the ICA through the CCA and ligated. The cervical skin was sutured to make 1-cm thread left in the outside. After 2 h of ischemia, the thread was removed and the skin was sutured after disinfection. A warm pad was performed to maintain the animal temperature at 37 °C during modeling. After MCAO surgery, tramadol (1 mg/kg) (Solarbio, Cat#YZ-171255) and penicillin sodium (15 mg/kg) (Solarbio, Cat#C8250) were injected for all rats to relieve pain and avoid infection. All experimental operations were performed from 8:00 am to 12:00 pm.

48 h after MCAO induction, rats with significant hemiplegia symptoms and neurological deficits were screened out [31]. Rats with similar neurological deficits were finally selected to maintain the reproducibility of the experimental results (Modified neurological severity score = 11 or 12) [32]. A total of 10 rats were anesthetized with an intraperitoneal injection of 1.2% pentobarbital sodium (40 mg/kg) and decapitated. A standard tissue block centered on the right anterior fontanel (± 2-mm) zone was extracted. Tissue block of the same location in control rats was obtained. The brain tissue was cut into pieces. Following addition of Dulbecco's Modified Eagle Medium (DMEM) at 150 mg/mL, magnetic beads were used to crush the tissue pieces. Centrifugation was performed 10,000*g* for 20 min at 4 °C, and 0.22-μm filter was used to obtain cerebral tissue extracts. The supernatant was reserved at − 80 °C for the treatment of UCMSCs.

### UCMSC culture and identification

The exosomes derived from UCMSCs were injected into rats or co-cultured with the HUVECs to clarify possible effect and mechanism of action of exosomes. UCMSCs and HUVECs were obtained from human umbilical cord. The informed consent from umbilical cord donors could not be made publicly available due to the regulations of Characteristic Medical Center of People’s Armed Police Forces. Briefly, neonatal cords were washed with sterile normal saline in a biosafety cabinet and then cut to 2-cm in size. The adventitia, umbilical artery, and vein were stripped, and the residual blood was cleared. Cords were then cut into patches at 1 mm^3^. The patches were then digested in a mixture containing 0.2% hyaluronidase and 0.2% collagenase type II at 37 °C. After 3 h, the mixture was filtered with a 200-mesh sieve to remove bulk tissues. Centrifugation was performed at 2000 rpm for 5 min, and the supernatant was discarded. Cells were cultured with culture medium, with half-volume of medium change every 2 days. CD90 (CD90 monoclonal antibody, 1:500 dilution, Cat#ab181469, Abcam) and CD105 (CD105 Rabbit monoclonal antibody, 1:500 dilution, Cat#ab231774 Abcam) were used to identify UCMSCs.

### Exosome extraction and identification

UCMSCs were firstly treated with cerebral infarcted tissue extracts for 24 h, followed by culture with exosome-free serum for 2 days. The supernatant was collected and centrifuged at 2000*g* for 20 min. The precipitates were discarded. The remaining fraction was filtered on a 0.22-μm sieve and then centrifuged successively at 10,000*g* (30 min) and 100,000*g* (70 min). The supernatant was discarded. The remaining precipitates were resuspended by sterile PBS. UCMSCs-derived exosomes were aliquoted and stored at − 80 °C.

Morphology and size of the exosomes were observed via transmission electron microscopy (TEM). Briefly, 10 μL of exosomes was absorbed onto a copper grid. After standing at room temperature for 2 min, 1% (W/V) phosphotungstic acids dye solution was dripped on the grid and the dye solution was absorbed after 5 min. The grid was dried under a lamp and then loaded to the TEM for observation. Exosomes were identified by expression of CD9 (CD9 polyclonal antibody, 1:500 dilution, Cat#PA5-85955, Invitrogen, RRID: AB_2802756) and CD63 (CD63 Monoclonal Antibody, 1:200 dilution, Cat#MA1-19281, Invitrogen, RRID: AB_1073284). Meanwhile, lipophilic tracer PKH26 (PKH26 Red Fluorescent Cell Linker Kit, Cat#PKH26PCL, Sigma, USA) was prepared for uptaking of exosomes by HUVECs. Exosomes derived from the UCMSCs were incubated with PKH26 at room temperature for 10 min. The fluorescence-labeled exosomes were incubated with HUVECs for 24 h. The uptake of PKH26 by the exosomes of HUVECs was visualized using confocal microscopy with 1 μg/mL Hoechst 33342 (Cat#H3570, Invitrogen) stain.

### Exosome injection

Rats were categorized into 5 groups at random: sham (*n* = 31), MCAO (*n* = 34), EXO (normal exosome, *n* = 35), N-EXO (exosomes from UCMSCs which are preconditioned by normal cerebral tissue extracts, *n* = 35), and I-EXO (exosomes from UCMSCs which are preconditioned by cerebral infarcted tissue extracts, *n* = 35). Seven rats died during the operation (MCAO group: 2, EXO group: 2, N-EXO group: 1, I-EXO group: 2). 6 rats were excluded from the experiment because their mNSS scores were below 10 (1 in MCAO, 1 in EXO, 2 in N-EXO, 2 in I-EXO). The experimental procedure is as follows:

80 μg exosomes was injected through the tail vein before removal of the surgical thread in MCAO rats. 0.01 M PBS of equal volume was injected in sham rats. Exosome injection was repeated on day 1 and 2 after operation.

### TTC staining and MRI

TTC staining was performed to observe the infarct size. Specifically, rats were anesthetized and then decapitated. The brain was extracted and frozen at − 20 °C for 20 min. The brain was sectioned transversely into 6 slices on a mold and then stained with 2% 2,3,5-triphenyl tetrazolium chloride (TTC, Cat#T8877, Sigma-Aldrich, USA) dye solution for 30 min in the dark. The sections were fixed in 4% paraformaldehyde for 1 h. The quantitative analysis of infarct volume was analyzed by calculation formula: Percentage of cerebral infarct volume = (sum of cerebral infarct volumes for each section) / (sum of volumes for each section) × 100%. On day 3 after MCAO induction, cranial magnetic resonance imaging (MRI) was performed. T2-weighted images (T2WI) were obtained to assess the infarct size and cerebral edema.

### Detection of neurological function

Modified Neurological Severity Scores (mNSS) and Morris water maze (Xmaze, Cat# XR-XM101, Shanghai XinRuan Information Technology) experiment were used to detect the neurological function of rat. The degree of neurological injury was assessed with mNSS. At days 1, 3, and 7 after MCAO induction, single-blind measure of neurological function was performed.

Before MCAO induction, water maze training was performed for 5 days in all rats to normalize the criteria. Before training, the platform was placed in the northeast quadrant and rats were put into the pool facing the pool wall. The swimming route of the rats was tracked by a camera. The escape latency was by definition the time to enter the water to climbing to the platform. Successful training was considered when the rats located the platform, climbing upon it and staying for at least 2 s. Rats that failed to find the hidden platform within 90 s were guided to the location of the platform and its escape latency was recorded as 90 s. Each rat was trained for 4 times in total. After the training, MCAO modeling and interventions were performed. Training was performed on days 3, 4, and 5 after operation, 4 times daily. On day 6, rats were subjected to cognitive testing in the form of space searching experiment. The escape latency, swimming speed, number of crossing the platform, and time in the target quadrant were recorded and analyzed.

### Immunofluorescence staining

For perfusions, rats were anesthetized through intraperitoneal injection of 1.2% pentobarbital sodium (40 mg/kg). Cardiac perfusion was conducted by using normal saline and 4% paraformaldehyde successively. On the following day, dehydration was obtained with 30% sucrose solution for another 5 days. Sections of brain tissues (25 μm thickness) were prepared in the coronal plane. Frozen sections transparent with 0.5% Triton X-100 and blocked with 10% normal goat serum (NGS). TdT solution and fluorescence-labeled dUTP solution (Click-iT Plus TUNEL Assay Kit, Cat#C10617, Invitrogen, USA) were mixed and incubated in the dark for 1 h. Hoechst 33342 was used for counterstaining. Tunel-positive cells were observed by fluorescence microscopy and counted by Image-J. Primary CD31 (CD31 polyclonal antibody, 1:50 dilution, Cat#PA5-32321, Invitrogen, RRID: AB_2549792) and Ki67 (Ki-67 Monoclonal Antibody, 1:100 dilution, Cat#14-5698-82, Invitrogen, RRID: AB_10854564) antibodies were dripped for overnight incubation at 4 °C and subsequent incubation with fluorescence-labeled secondary antibody (Goat anti-Rabbit Alexa Fluor 594, Cat#A-11037, Invitrogen, RRID: AB_2534095; Goat anti-Rat Alexa Fluor 488, Cat#A-11006, Invitrogen, RRID: AB_2534074) for 4 h in the dark. Hoechst 33342 was used for counterstaining. Slides were prepared to observe vascular remodeling. The sections were observed under a confocal microscope (Nikon A1R, Nikon, Japan).

### Exosome miRNA sequencing

QIAseq library building method of the QIAseq miRNA Library Kit was used to constructe stable miRNA-specific libraries. The Illumina's Hiseq 2500 sequencing platform was used to perform high-throughput sequencing. Expression difference analysis was performed on miRNA significant differential expression profiles. GO functional enrichment and KEGG pathway analysis were performed on the differential miRNA target genes. Basing on grouping information, we can identify molecular function (MF), biological process (BP) and cellular component (CC) that are significantly associated with miRNA differential expression profile target genes. The molecular signaling pathways that are enriched for differentially expressed miRNA target genes also can be analyzed.

### Oxygen–glucose deprivation/reoxygenation and treatment

Human umbilical vein endothelial cells (HUVECs) which are obtained from human umbilical cord were used to mimic cerebrovascular endothelial cells, and the treatment of OGD/R was performed to mimic cerebral ischemia reperfusion (I/R). Morphology and growth status of HUVECs were identified under a phase-contrast microscope. vWF (vWF Polyclonal Antibody, 1:200 dilution, Cat#PA5-16634, Invitrogen, RRID: AB_10982615) and CD31 were used to identify HUVECs. Procedures for OGD/R induction were as follows. HUVECs were cultured with glucose-free medium in the surroundings containing 95% N_2_ and 5% CO_2_ at 37 °C for 12 h. Reoxygenation condition was induced by recover of glucose and oxygen. HUVECs were cultured at 37 °C with 5% CO_2_ for 24 h as reoxygenation.

HUVECS with similar density were subjected to simple randomization by QuickCalcs from GraphPad. The experiments were repeated at least three times for each experiment, unless stated otherwise. Four groups were generated: control group, EXO group (normal exosomes), N-EXO group (exosomes from UCMSCs which are preconditioned by normal cerebral tissue extracts), and I-EXO group (exosomes from UCMSCs which are preconditioned by cerebral infarcted tissue extracts). Exosomes were used at 0, 20, 40, and 60 μg/mL for 24 h before reoxygenation. Cell morphology was monitored via digital holographic microscopy (DHM). CCK8 kit was used to detect cell viability. The OD values were detected at 450 nm by a microplate reader. The ratio of cell viability was used to detect the therapeutic effect of pretreatment exosomes. Scratch healing assay was performed to examine cell migration. Cell migration was observed every 12 h under an inverted phase contrast microscope and images were captured.

### Western blotting

HUVECs were randomly divided into Control, OGD, EXO, N-EXO, and I-EXO groups. Expression of Bax, Bcl-2, and Caspase-3 in each group was measured. Cells were lysed with radio immunoprecipitation assay (RIPA) and phenylmethylsulfonyl fluoride (PMSF) (100:1) on ice. Protein extracts were denatured and subjected to 10% sodium dodecyl sulfate polyacrylamide gel electrophoresis (SDS-PAGE). The proteins were transferred to a 0.22-μm polyvinylidene fluoride (PVDF) membrane, which was then blocked in 5% skim milk. Primary antibodies against Bax (Bax Monoclonal Antibody, 1:1000 dilution, Cat#MA5-14003, Invitrogen, RRID: AB_10979735), Bcl-2 (Bcl-2 Polyclonal Antibody, 1:1000 dilution, Cat#PA5-27094, Invitrogen, RRID: AB_2544570), and Caspase-3 (Caspase 3 Monoclonal Antibody, 1:1000 dilution, Cat#MA1-16843, Invitrogen, AB_568479) were hybridized at 4 °C overnight. The membranes were incubated with horseradish peroxidase-conjugated anti-rabbit (Goat anti-Rabbit IgG (H + L) Secondary Antibody, Cat#31460, Invitrogen, RRID: AB_228341) or anti-mouse (Goat anti-Mouse IgG (H + L) Secondary Antibody, Cat#31430, Invitrogen, RRID: AB_228307) secondary antibodies. ECL was applied to develop protein bands.

### Statistical analysis

GraphPad Prism 7.0 software (RRID:SCR_002798) was used to perform data processing and analysis. Data acquisition and analyses were blinded to the experimenter. One Sample Kolmogorov–Smirnov test was performed to determine normal distribution for all data in this study. Measurement data in mean ± standard deviation were reported. Statistical significance was determined by one-way analysis of variance (ANOVA) which is followed by Bonferroni analysis in multiple groups. Two-tailed student’s t was performed for pairwise comparisons. *P* < 0.05 was considered statistically significant.

## Results

### Identification of UCMSCs-derived exosome

**Fig. 1:**
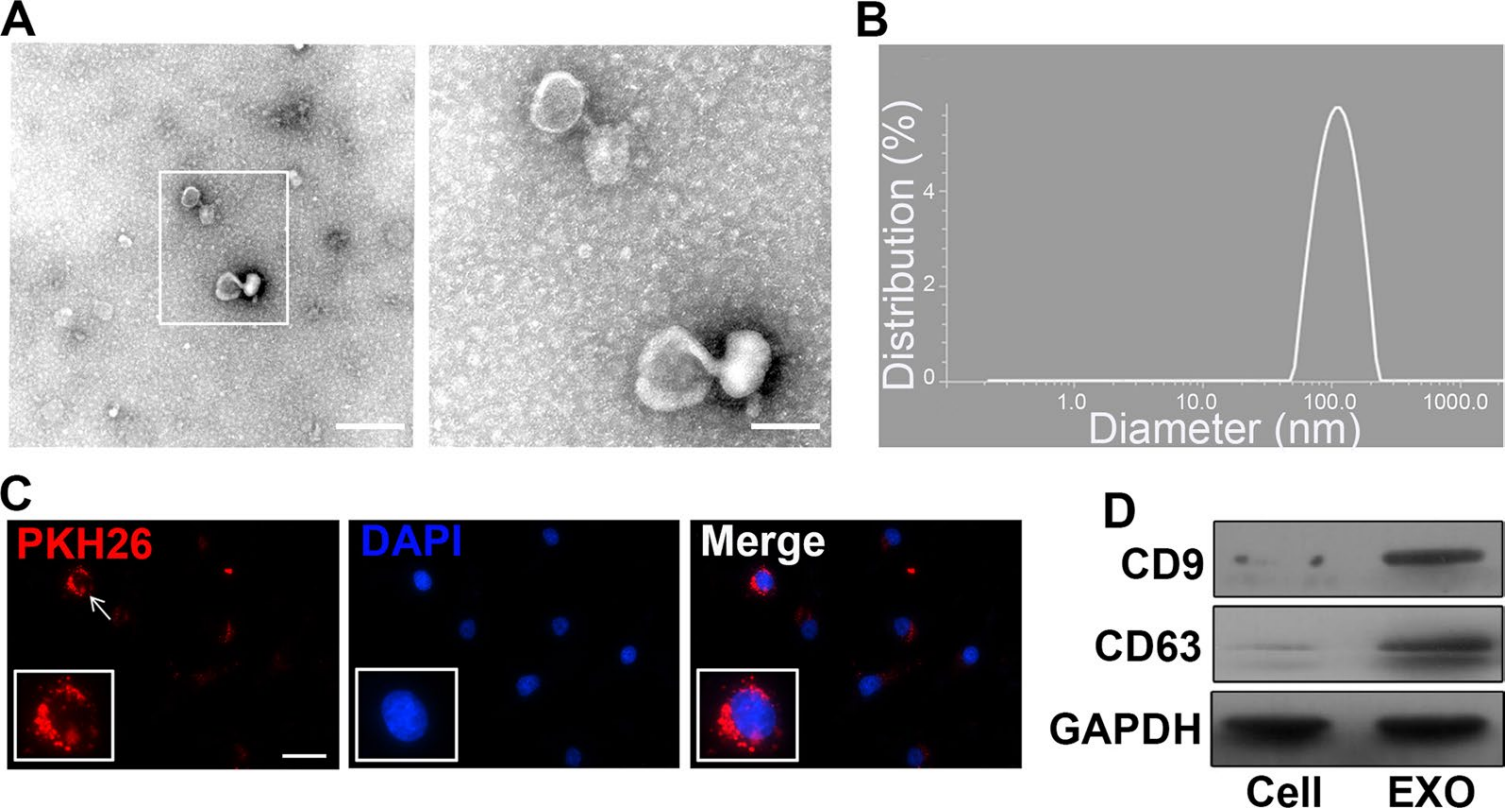
Exosome characterization and demonstration that exosomes are derived from infarct-preconditioned UCMSCs can be taken up by HUVECs. **A** Transmission electron microscopy images of exosomes. **B** Measurement of the diameter of exosomes. **C** Infarct-preconditioned exosomes labeled with PKH26 emitted red fluorescence. **D** Representative Western blots confirmed the expression of CD9 and CD63, which are markers of exosomes. Scale bars in A represent 250 nm (left) and 100 nm (right) and in C represent 50 μm

Identification of UCMSCs and HUVECs from human umbilical cords by specific maker. (CD90 and CD105 for UCMSCs (Additional file 1: Fig. S2A-C), vWF and CD31 for HUVECs (Additional file 1: Fig. S2D-F)). The TEM results revealed that the diameter of pellets was between 50 and 120 nm. A bilayer structure was observed, which was in a saucer-cup disk-like shape or in oval (Fig. 1A). The peak particle size of the pellets was 98 nm (Fig. 1B). Exosomal specific protein markers CD9 and CD63 exhibited positive expressions with Western blotting (Fig. 1D). Nuclei with peripheral red exosomes (PKH26) were revealed under confocal laser microscopy (Fig. 1C). These results inferred that HUVECs have the ability to take up exosomes derived from UCMSCs.

### Preconditioned exosomes reduced brain damage volume after stroke

**Fig. 2:**
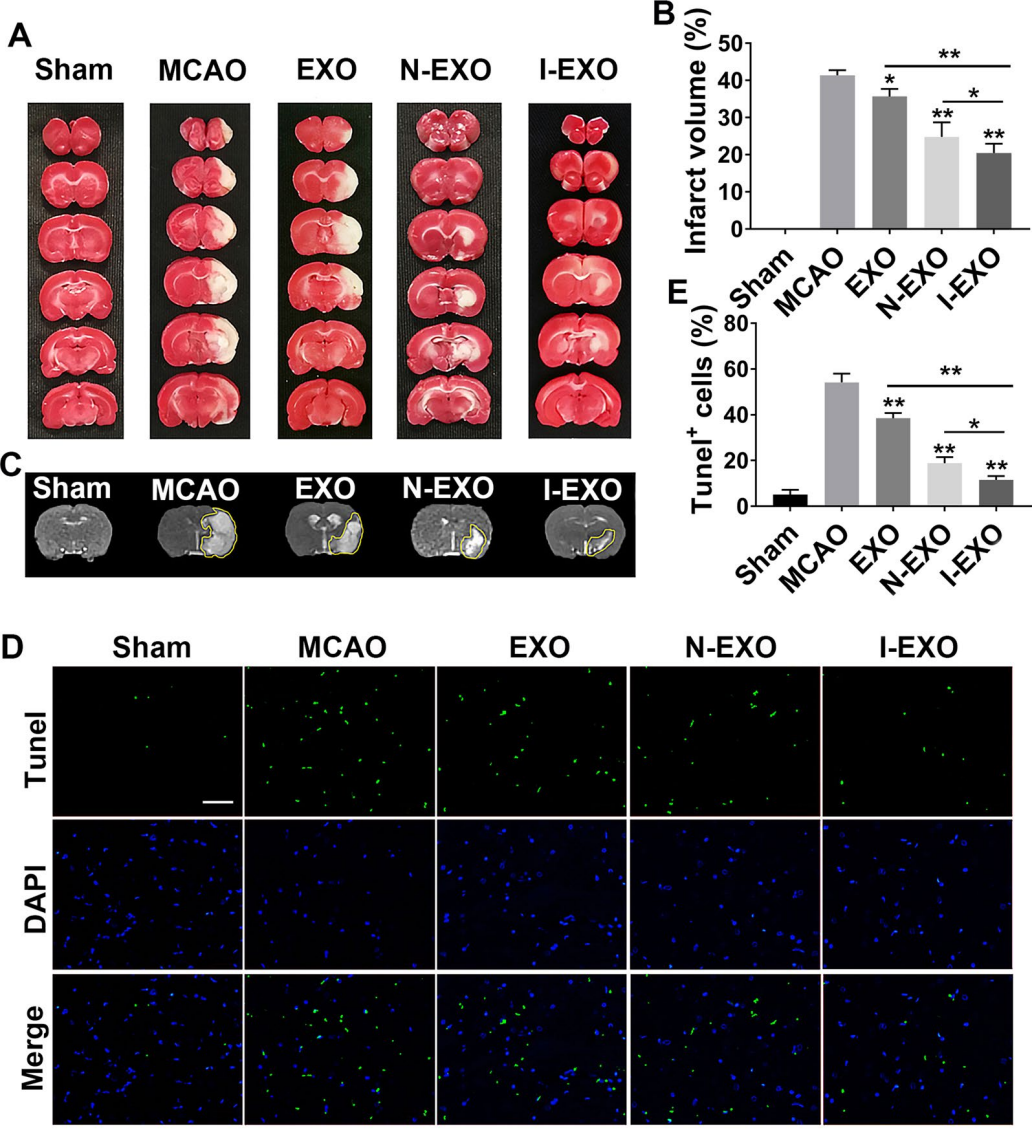
Infarct-preconditioned exosomes reduced brain damage volume and neural apoptosis after stroke. **A** TTC-stained representative images of brain sections after middle cerebral artery embolism (MCAO). **B** Representative images of cranial magnetic resonance imaging (MRI) after MCAO. **C** Apoptotic cells labeled with Tunel emitted green fluorescence. **D** Quantification of the percentage of Tunel^+^ cells in differentiated exosomes injection after MCAO. **E** Quantitative analysis of infarct volume after MCAO. *P < 0.05, **P < 0.01 versus MCAO group and EXO group. Scale bars in C represent 200 μm. *n* = 3 in each group

TTC staining showed bright red in the healthy side of brain sections from MCAO rats while pale in the diseased side. The infarct volume was more than 40% after MCAO (41.39 ± 0.7992%). Normal exosomes injection after MCAO decreased the infarct volume (35.69 ± 1.188%, *P* < 0.05). Compared to N-EXO (exosomes from UCMSCs which are preconditioned by normal cerebral tissue extracts group, I-EXO (exosomes from UCMSCs which are preconditioned by cerebral infarcted tissue extracts) treatment significantly reduced the infarct volume (20.8 ± 1.257 *vs*. 26.69 ± 2.138%, *P* < 0.05) (Fig. 2A, B).

There was normal signal in sham group demonstrating absence of infarction by cranial MRI (T2WI). After induction by MCAO, the lesion hemisphere showed high signal with a clear border to the surrounding tissue. Treatment with EXO and N-EXO (exosomes from UCMSCs which are preconditioned by normal cerebral tissue extracts) led to decreasing degree of edema. A more significant decrease in the degree of edema was detected in I-EXO group (Fig. 2C).

### Preconditioned exosomes decreased neural apoptosis after stroke

Neural apoptosis is one of the major pathological changes in brain after stroke. We explored the effect of exosome on neural apoptosis after MCAO. TUNEL staining was performed to identify apoptotic cells. Compared with the sham group, MCAO induction led to cerebral infarction with a substantial increase in Tunel positive cells (54.14 ± 1.917 *vs*. 5.152 ± 1.047%). The number of apoptotic cells was decreased when exosomes were applied (38.49 ± 1.142% in EXO and 18.82 ± 1.336% in N-EXO, *P* < 0.01). And the Tunel positive cells in I-EXO group had a smaller number compared to N-EXO group (11.56 ± 0.8268% *vs*. 18.82 ± 1.336%, *P* < 0.05). These results suggested that infarct-preconditioned exosomes reduce neural apoptosis and promote neuron survival (Fig. 2D, E).

### Preconditioned exosomes improved neurological function after stroke

**Fig. 3:**
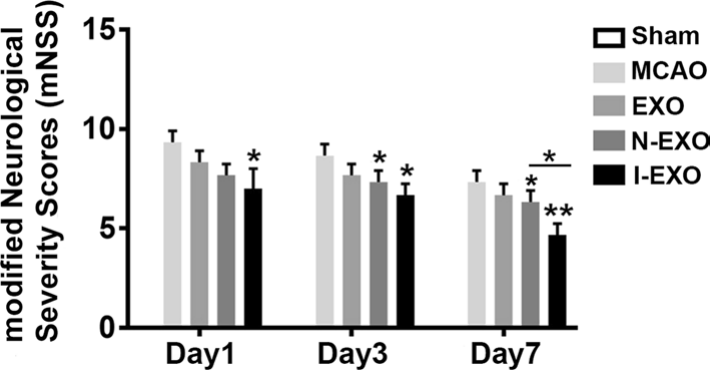
Infarct-preconditioned exosomes improved neurological function after stroke. Rat mNSS score was recorded at 1, 3, 7 day in differentiated exosomes injection after MCAO. *P < 0.05, **P < 0.01 versus MCAO group and EXO group. *n* = 9 in each group

We next explored whether injection of exosomes could improve motor functional recovery and rescue brain damage. mNSS method was introduced to evaluate rat neurological function on day 1, 3, and 7 post-MCAO. The highest mNSS score occurred in the MCAO group and gradually subsided with time. Decreased mNSS score was found in EXO, N-EXO, and I-EXO group as compared to MCAO group. Notably, I-EXO (4.625 ± 1.060) treatment recovered neurological function to higher level, wherein no difference was found between EXO (7.5 ± 1.195 *vs*. 4.625 ± 1.060, *P* < 0.05) and N-EXO groups (6.75 ± 0.886 *vs*. 4.625 ± 1.060, *P* < 0.05) (Fig. 3).

**Fig. 4:**
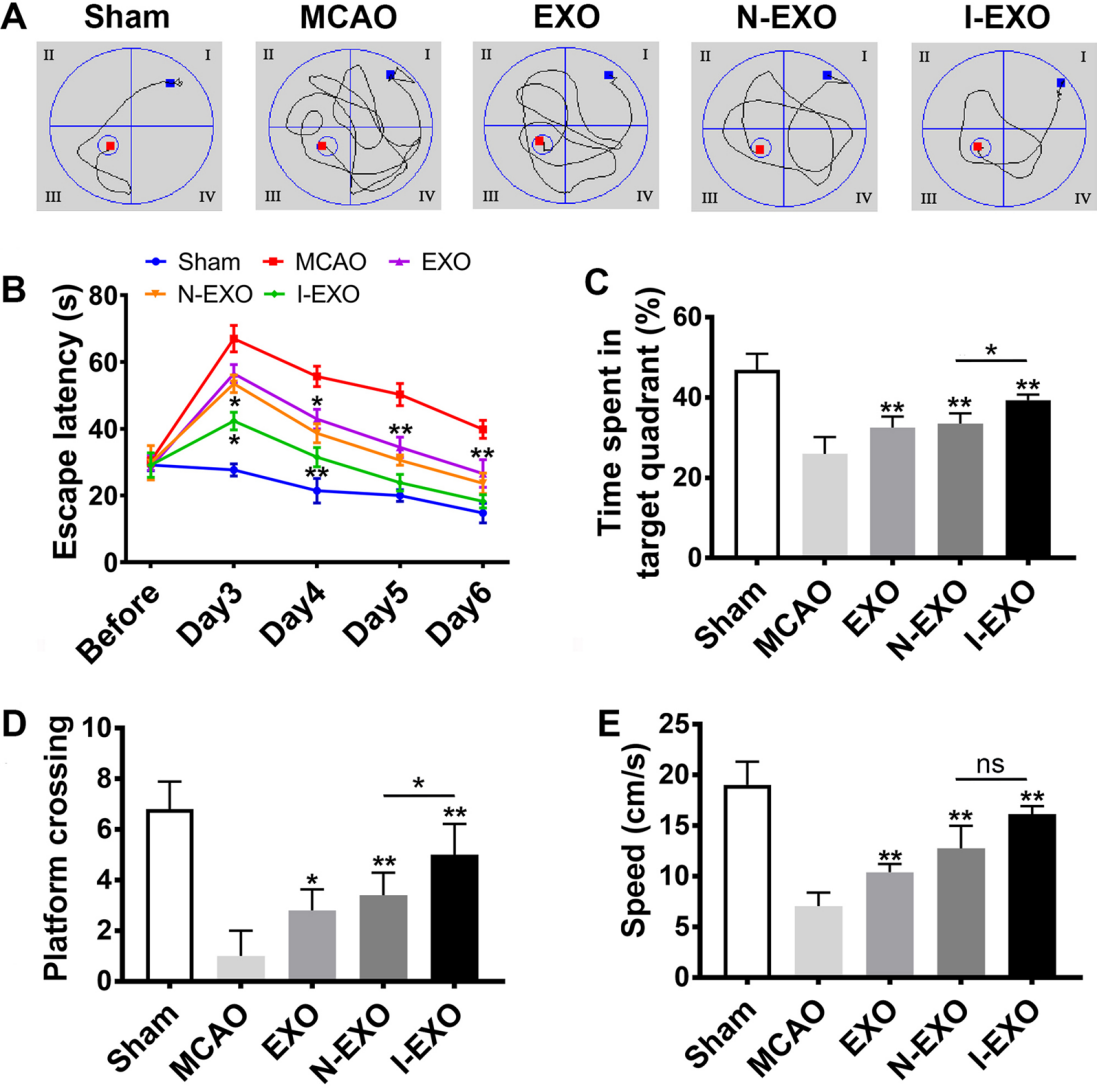
Infarct-preconditioned exosomes improved spatial learning and memory ability after stroke. The rats were trained to remember the hidden platform in the maze from 3 to 6 days after MCAO. **A** Representative routes of each group. **B** The latency was recorded. **C** The time stayed in the target quadrant of each group. **D** The number of platform crossings of each group. **E** Swimming speed of each group. *P < 0.05, **P < 0.01 versus MCAO group and EXO group. *n* = 10 in each group

Morris water maze test was used to assess whether preconditioned exosomes could impart any cognitive benefits. Overall there is improvement in spatial memory ability in MCAO rats after exosomes treatment with shortening of searching routes (Fig. 4A). The MCAO group exhibited a notable cognitive defect compared to the sham group. The EXO group promoted cognitive by decreasing latency, increasing the time spent in target quadrant and the number of platform crossings compared with the MCAO group (*P* < 0.05) (Fig. 4B–D). It is noteworthy that the I-EXO group compared to the N-EXO group showed a higher time spent in target quadrant (39.725 ± 1.131% *vs.* 30.878 ± 1.898%, *P* < 0.05) and more platform crossings (5.167 ± 1.602 *vs.* 3.833 ± 1.329, *P* < 0.05). The swimming speed was also increased in the EXO group (Fig. 4E). However, administration of exosomes preconditioned with infarcted brain extract in the I-EXO group amplified the improvement in cognitive recovery compared to that in the N-EXO group (*P* < 0.05) (Fig. 4B–E). These results suggested that infarct-preconditioned exosomes have a further protective effect on MCAO-induced cognitive impairment.

### Preconditioned exosomes promoted cerebrovascular remodeling after stroke

**Fig. 5:**
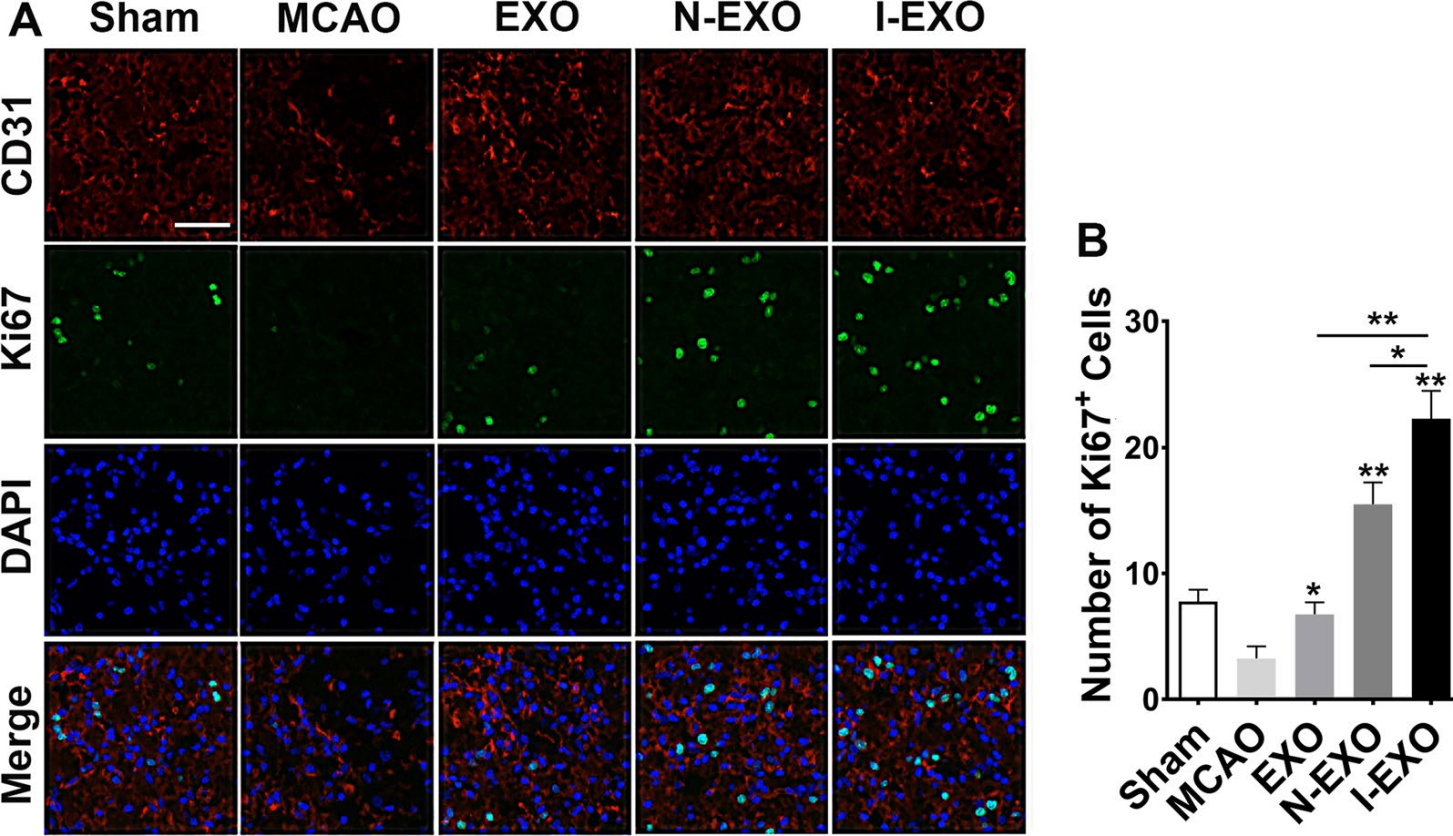
Infarct-preconditioned exosomes promoted vascular remodeling after stroke. **A** Representative images of vascular remodeling by immunostaining of new formed blood vessels (Ki67^+^/CD31^+^) in the rat infarction periphery after MCAO. Ki67, proliferation marker (green); CD31, endothelial marker (red); DAPI labels nuclei (blue). **B** Quantitative analysis of Ki67^+^/CD31^+^ new formed blood vessels in each group. *P < 0.05, **P < 0.01 versus MCAO group, EXO group and N-EXO group. *n* = 3 in each group

As I-EXO treatment significantly decreased infarct volume and improved neurological function after stroke, we further explored the mechanisms underlying I-EXO treatment after MCAO. We observed vascular remodeling by co-immunostaining of cell proliferating marker Ki67 and endothelial cell markers CD31 after 7 days of MCAO induction (Fig. 5A). As compared with the MCAO group, more cells positive for both Ki67 and CD31 were observed in the EXO and N-EXO groups (*P* < 0.05). Additionally, the degree of vascular remodeling in I-EXO group was relatively higher than that in EXO and N-EXO group (*P* < 0.05) (Fig. 5B).

### Screening of exosomal differentially expressed miRNAs and analysis of target genes

We normalized the raw data into log_2_ values and plotted a scatter plot in a two-dimensional coordinate system. There were 236 differentially expressed miRNAs between EXO and I-EXO groups. 139 miRNAs were up-regulated, which including hsa-miR-211-3p, hsa-miR-181a-5p, hsa-miR-148a-3p were up-regulated. Besides, there were 214 differentially expressed miRNAs between N-EXO and I-EXO groups, among which 137 miRNAs such as hsa-miR-124-3p, hsa-miR-128-3p, hsa-miR-132-3p were up-regulated (Data not shown).

**Fig. 6:**
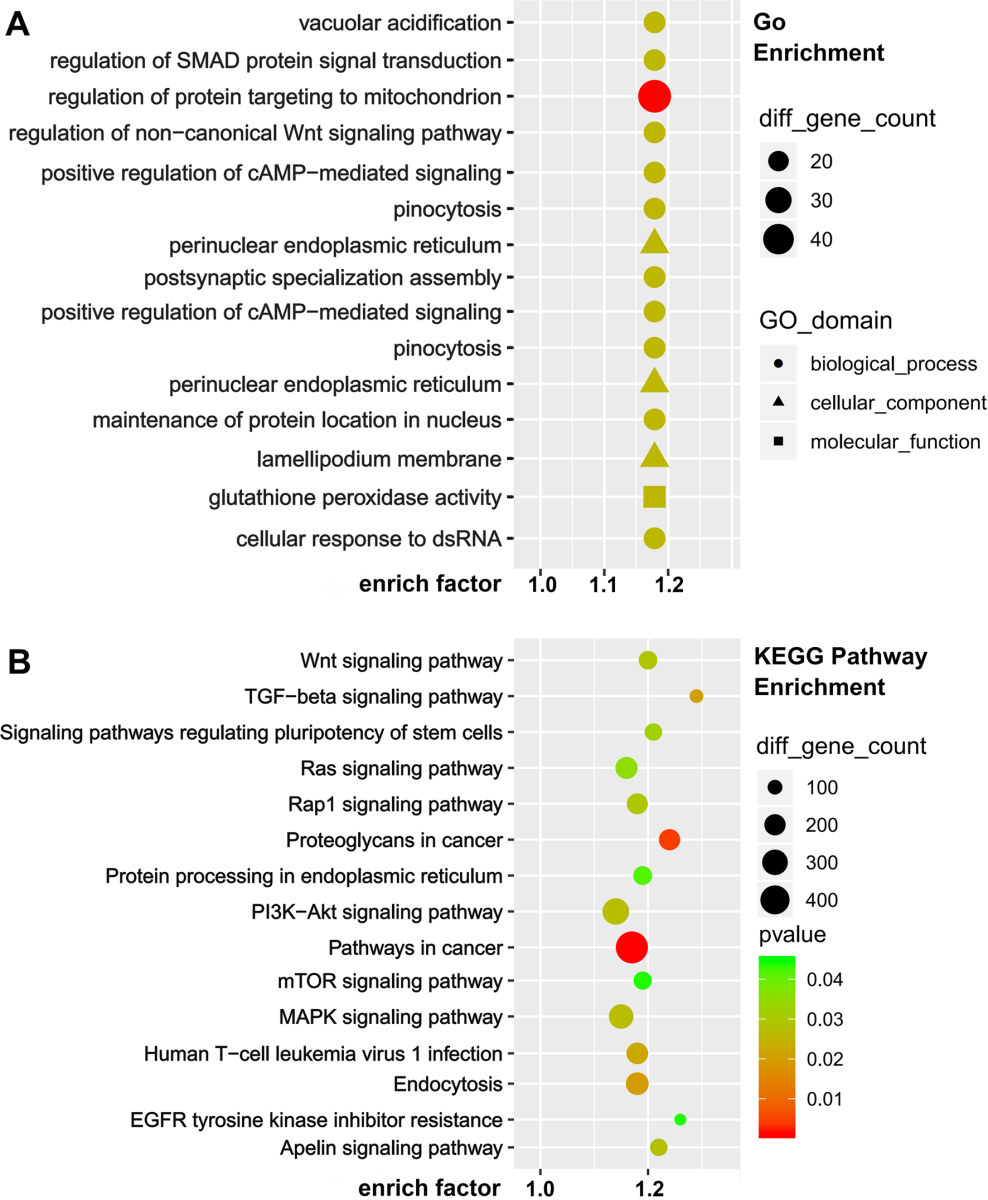
Enrichment analysis identified miRNA target genes involved in altered pathways following infarct preconditioning. **A** GO: term molecular functions and biological process involved within the observed miRNAs. **B** KEGG pathway analysis identified significantly enriched pathways involved within the infarct preconditioning

We performed GO analysis of target genes and found that the biological process genes are mainly distributed in biological regulation, cellular process, single-organism process. The cellular component genes are mainly distributed in cell, cell part, organelle, and the molecular function genes are mainly distributed in binding, catalytic activity. We then analyzed the first 30 genes enriched by GO. I-EXO mainly affected the regulation of protein targeting to mitochondrion, the regulation of protein oligomerization, and perinuclear endoplasmic reticulum (Fig. 6A). These results suggested that the protective effects of infarct-preconditioned exosomes may be achieved by enhancing oxygen utilization and improving energy metabolism which is linked to the vascular remodeling. KEGG pathway analysis showed that the target genes were mainly enriched in signal transduction including PI3K-Akt signaling pathway, MAPK signaling pathway, mTOR signaling pathway, and TGF-β signaling pathway. KEGG enrichment suggested that I-EXO might regulate cell proliferation and apoptosis, and cellular glucose metabolism, which in turn ameliorates neurological impairment due to infarction (Fig. 6B).

### Preconditioned exosomes enhanced cell viability, reduced cell apoptosis

**Fig. 7:**
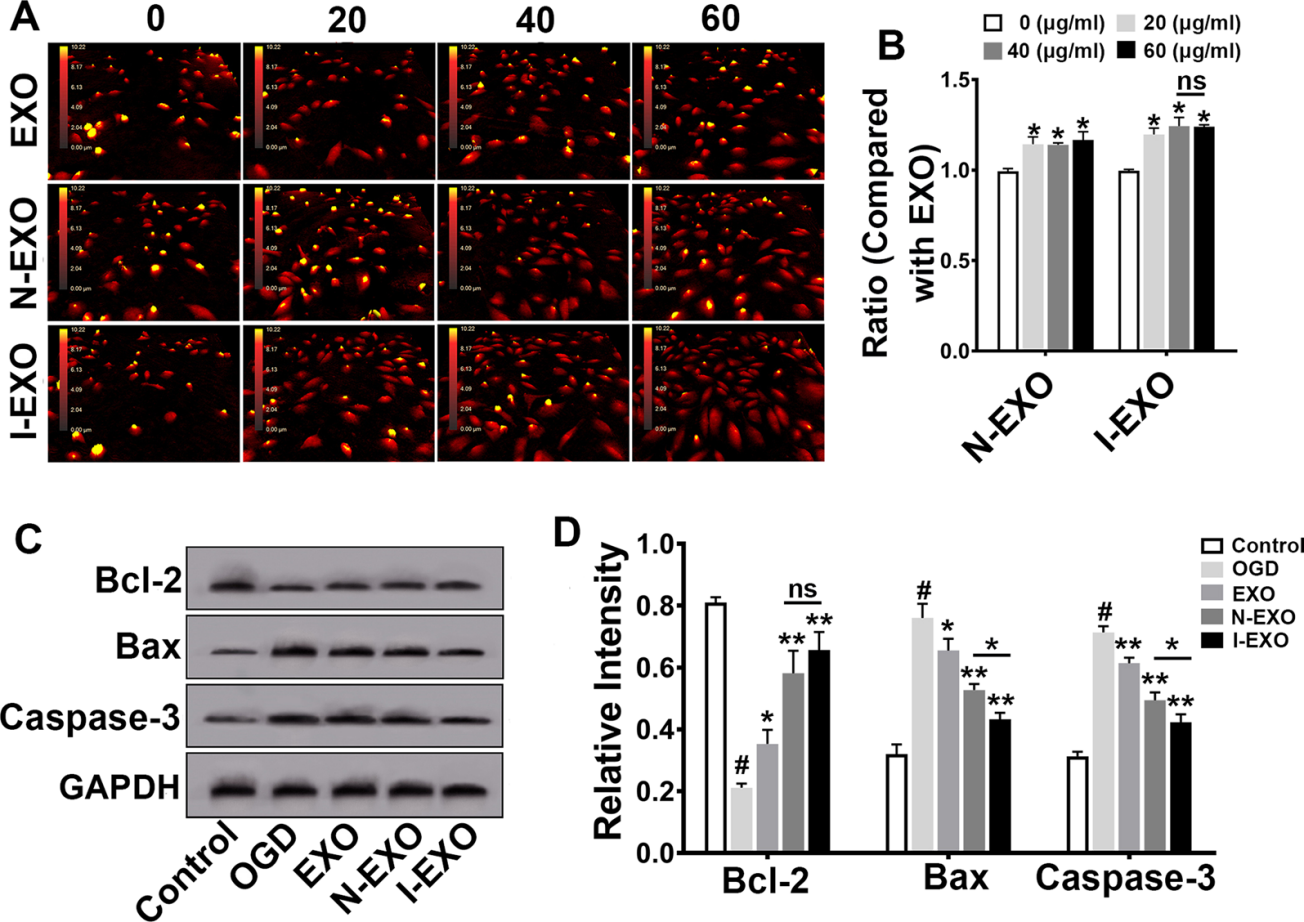
Infarct-preconditioned exosomes inhibited apoptosis of human umbilical vein endothelial cells (HUVECs) after oxygen–glucose deprivation/reoxygenation (OGD/R). **A** Representative images of cellular morphology of each group at 24 h after OGD/R by digital holographic microscopy (DHM). **B** The cell viability of N-EXO and I-EXO group. All data were obtained by ratio to normal exosomes. *P < 0.05 versus 0 (μg/mL) group. *n* = 3 in each group. **C** Expression levels of Bcl-2, Bax, and Caspase3 in HUVECs after OGD/R were examined by Western blotting. **D** Quantitative analyses of (**C**). *P < 0.05, **P < 0.01 versus OGD group and EXO group, ^#^*P* < versus control group. *n* = 3 in each group

To study the effect of preconditioned exosomes on vascular endothelial cells, we examined the apoptosis of HUVECs. HUVECs gradually became round and turned into golden yellow from previous red in DHM after 24 h of OGD/R. DHM presentations showed that compared with normal exosomes, HUVECs gradually became stretched and flatted with the intervention of infarct-preconditioned exosomes. The intercellular space was gradually narrowed. Meanwhile, the number of cells was increased (Fig. 7A). As shown by CCK-8 assay, I-EXO treatment markedly increased the viability of HUVECs compared with EXO group (*P* < 0.05). There is no statistical difference in the I-EXO groups when exosomes were applied at 40 μg/ml and 60 μg/ml (Fig. 7B).

Western blotting results (Fig. 7C) showed that OGD/R induced a higher expression of Bax and Caspase-3, while lower expression of Bcl-2 (OGD *vs*. Control, *P* < 0.05). Exosomes significantly attenuated the expression of Bax and Caspase-3 (EXO *vs*. OGD, I-EXO *vs*. OGD, *P* < 0.01) and increased the expression of Bcl-2. Interestingly, the I-EXO group showed the most remarkable change in Bax and Caspase-3 (I-EXO *vs*. N-EXO, *P* < 0.05) (Fig. 7D).

### Preconditioned exosomes improved the migration of HUVECs

**Fig. 8:**
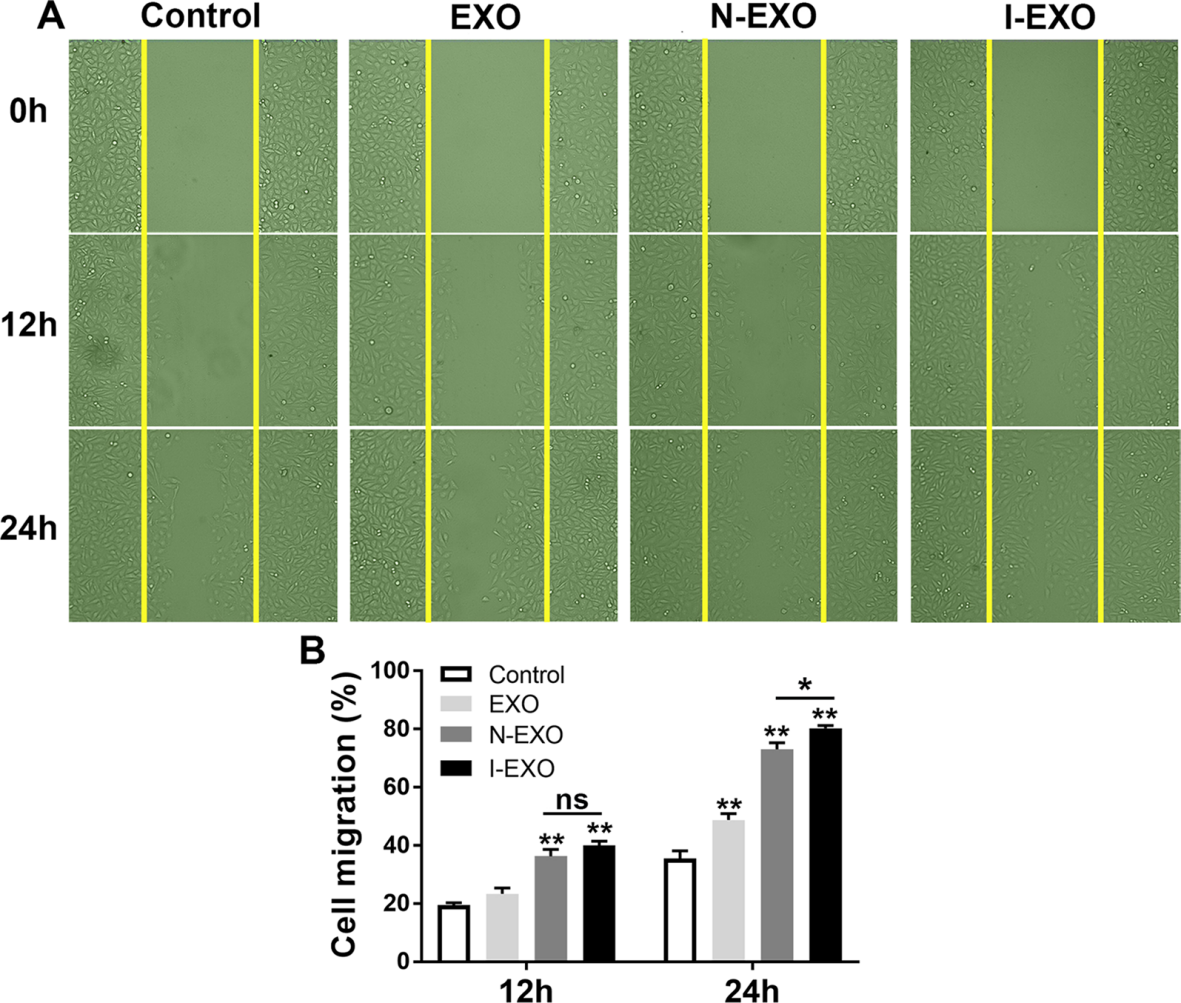
Infarct-preconditioned exosomes promoted migration of HUVECs. **A** Representative images of cell migration in each group at 0, 12, and 24 h after scratch healing. **B** Quantitative analyses of (**A**). **P < 0.01 versus Control group, *P < 0.05 versus EXO group. *n* = 3 in each group

To further validate that infarct-preconditioned exosomes contribute to vascular remodeling, we used scratch healing assay to test cell migration. Baseline scratch width was basically consistent among the control, EXO, N-EXO, and I-EXO groups. HUVECs began migrating toward the scratch after 12 h. Comparatively, the migration in the EXO, N-EXO, and I-EXO groups was higher compared to the control group at 24 h (*P* < 0.01) (Fig. 8A). The scratch healing area in the I-EXO group was significantly higher than that in the N-EXO groups (*P* < 0.05) (Fig. 8B).

## Discussion

Ischemic stroke has become the second leading cause of death worldwide [1]. The repair and remodeling of cerebrovascular endothelial cells play a key role in the treatment of ischemic stroke. MSCs exert injury repair by responding to the microenvironment of brain infarction. MSCs can promote vascular remodeling and neural repair by regulating the microenvironment around the injured tissue after reaching the injury area [33, 34]. Exosomes of MSCs might be the ideal alternative treatment option [35]. Exosomes have been shown to play the same role as the cells which are derived from in the repair process of a variety of tissues and organs [16, 36]. Therefore, it can be hypothesized that infarct-preconditioned exosomes can play a crucial role in vascular remodeling.

Exosomes are membranous structures with a bilayer containing abundant active components such as proteins, lipids, and RNA [37, 38]. Besides, exosomes are low immunogenic and non-tumorigenic and play an important communication role between cells [39]. In the current study, we found that exosomes isolated from UCMSCs can be taken up by cultured HUVECs. Several studies have shown that factors in exosomes have a strong influence on the survival of vascular endothelial cells [18, 40]. Exosomes derived from the infarct-like microenvironment can alter the fate of endothelial cells around injured area. Therefore, in order to investigate the function of exosomes from the infarct-like microenvironment, UCMSCs were preconditioned using infarcted cerebral tissue extracts.

Reperfusion time and infarct extent are key factors in determining the degree of neurological impairment after the onset of cerebral infarction. It was observed that infarct-preconditioned exosomes significantly reduced apoptosis in brain, and the effect was stronger than normal exosomes. In addition, the extent of cerebral infarction was further reduced by preconditioned exosomes. To evaluate the effect of infarct-preconditioned exosomes on neurological function after stroke, mNSS and Morris water maze were conducted. The results revealed that the administration of the infarct-preconditioned exosomes could significantly improve spatial learning and memory post-stroke compared with the effects of normal exosome administration. Infarct-preconditioned exosomes could significantly reduce mNSS scores and further improve the neurological functional parameters which included motor, sensory, and balance in rats. The results of our previous study showed that traumatic injury-preconditioned secretome could significantly improve cognitive function after TBI. And the increased benefits of secretome administration were attributable to the up-regulated molecules from the MSC secretome preconditioned by a traumatically injured microenvironment such as vascular endothelial growth factor, ciliary neurotrophic factor, and brain-derived neurotrophic factor [31]. Therefore, it is necessary to investigate the potential contribution of vascular remodeling to the recovery of neurological function after stroke.

Hypoxic and toxin-accumulating microenvironment leads low neonatal cell survival and maturation, while appropriate vascular endothelial remodeling is beneficial for neuronal survival. Our results showed a higher number of co-labeled CD31 and Ki67 in preconditioned exosomes group compared with normal exosomes group. Exosomes from MSC promoted functional recovery, at least in part, via promoting endogenous angiogenesis after traumatic brain injury [41]. Similarly, our results indicated that infarct-preconditioned exosomes could ameliorate cerebral infarction caused neurological damage. It has been shown that HIF-1α overexpression in MSCs cultured under hypoxic conditions induces enhanced activity of MSCs, as well as enhanced angiogenesis by the resulting secreted exosomes [34]. Some previous results confirmed that exosomes released from educated mesenchymal stem cells accelerated cutaneous wound healing via promoting angiogenesis [42]. In fact, restorative strategies after stroke are more focused on the remodeling of cerebral vascular endothelial cells.

To further validate the vascular remodeling ability of the preconditioned exosomes, miRNA sequencing was used to predict the biological functions of differentially expressed miRNAs. Among the genes upregulated in preconditioned exosomes, miR-181 was shown to promote vascular smooth muscle proliferation and to regulate the vascular smooth muscle phenotype in the microenvironment [43]. Similar results confirmed that miR-181b-5p can be transferred to vascular endothelial cells via exosomes and dramatically induced angiogenesis by targeting PTEN [44]. It has also been demonstrated that the downregulation of miR-211 will lead to vascular calcification and restoration of miR-211 can alleviate the calcification [45]. Moreover, further GO and KEGG enrichment analysis suggested that preconditioned exosomes regulated cell proliferation, endocytosis, and glucose metabolism. Meanwhile, target genes were highly enriched in PI3K-Akt, MAPK, and mTOR pathway. One published study confirmed that hypoxia-preconditioned NSC-derived exosomal miRNAs are associated with signaling pathways such as PI3K-Akt, Hippo, MAPK, mTOR, and endocytosis [46]. Several other studies have shown that miRNAs in exosomes vary in different periods of stroke [47]. Results from the current study suggested that the infarct-like microenvironment can result in exosomes containing more miRNAs that promote vascular remodeling.

HUVECs were used to mimic cerebrovascular endothelial cells in order to better validate the effect of infarct-preconditioned exosomes in vitro. Significant improvement in cell viability and morphology after treatment with preconditioned exosomes was observed compared to normal exosomes. Infarct-preconditioned exosomes further decreased the expression level of Bax and Caspase-3 and increased Bcl-2 activation, thus reducing the damaging effect for HUVECs caused by OGD/R. These results indicated that the infarct-preconditioned exosomes further improved the pro-apoptotic environment after stroke to reduce apoptosis, which may be the key reason of the infarct-preconditioned exosomes to promote cerebrovascular remodeling. Infarct-preconditioned exosome group showed more pronounced cell migration rate and healing of the cell scratch area compared to normal exosome group. All these results suggested that infarct-preconditioned exosomes enhanced the directional migration of HUVECs and reduced apoptosis caused by OGD/R.

## Conclusion

In summary, our findings confirmed that infarct-preconditioned exosomes enhanced post-stroke recovery through promoting vascular remodeling, which can be considered as a new alternative strategy.

## Supplementary Information

**Additional file 1.** Identification of UCMSCs/HUVECs and time-line of experimental procedure.

## Data Availability

Not applicable.

## Abbreviations

UCMSC: Umbilical cord mesenchymal stem cell
MCAO: Middle cerebral artery occlusion
HUVEC: Human umbilical vein endothelial cell
OGD/R: Oxygen–glucose deprivation/reoxygenation
BBB: Blood–brain barrier
IL-6: Interleukin-6
VEGF: Vascular endothelial growth factor
TBI: Traumatic brain injury
RIPC: Remote ischemic preconditioning
DNMT3B: DNA-methyltransferase 3 beta
MALAT1: Metastasis-associated lung adenocarcinoma transcript 1
CCA: Common carotid artery
ICA: Internal carotid artery
mNSS: Modified Neurological Severity Score
TEM: Transmission electron microscopy
TTC: 2,3,5-Triphenyl tetrazolium chloride
MRI: Magnetic resonance imaging
MWM: Morris water maze
NGS: Normal goat serum
GO: Gene ontology
KEGG: Kyoto Encyclopedia of Genes and Genomes
SD: Sprague–Dawley
MF: Molecular function (MF)
BP: Biological process
CC: Cellular component
I/R: Ischemia reperfusion
DHM: Digital holographic microscopy
RIPA: Radio immunoprecipitation assay
PMSF: Phenylmethylsulfonyl fluoride
SDS-PAGE: Sodium dodecyl sulfate polyacrylamide gel electrophoresis
PVDF: Polyvinylidene fluoride
Bcl-2: B-cell lymphoma 2
MSC: Mesenchymal stem cell
PI3K: Phosphatidylinositol 3-kinase
MAPK: Mitogen-activated protein kinase
mTOR: Mammalian target of rapamycin
