# Supplementary material for: Infarct-preconditioning exosomes of umbilical cord mesenchymal stem cells promoted vascular remodeling and neurological recovery after stroke in rats
Source: Stem Cell Res Ther. 2022 Jul 28;13:378. doi: 10.1186/s13287-022-03083-9 (PMC9330700; doi:10.1186/s13287-022-03083-9)
Supplement: Supplementary file 1 — Additional file 1. Identification of UCMSCs/HUVECs and time-line of experimental procedure. [file 13287_2022_3083_MOESM1_ESM.docx]

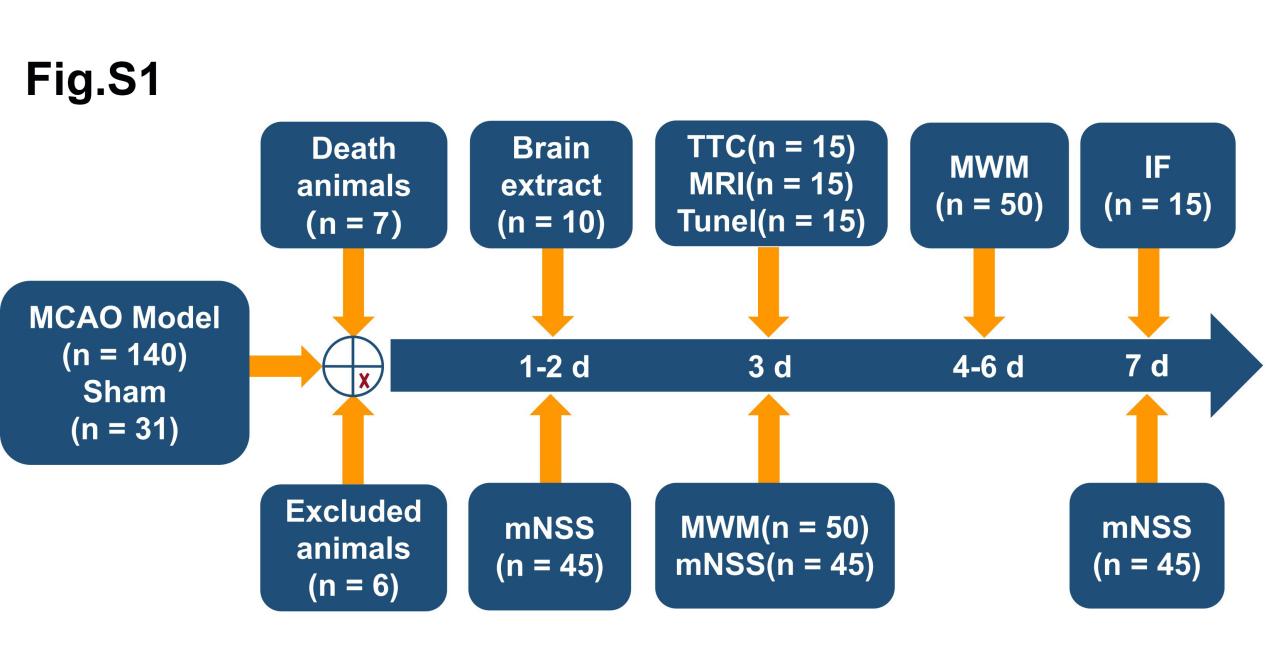


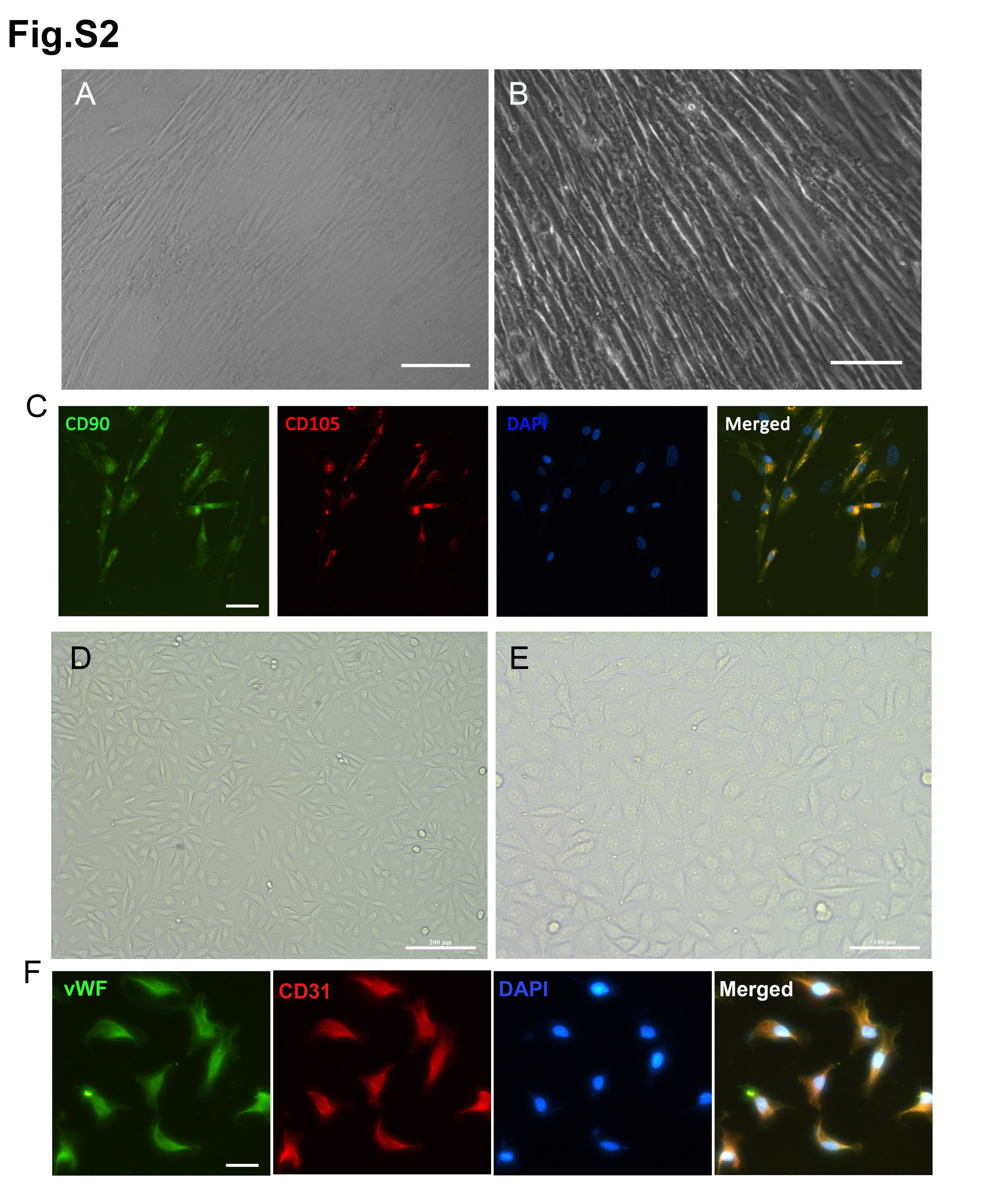


**Figure Legend**

Fig. S1 Time-line of experimental procedure. All operations were performed from 8:00 am to 12:00 am in 140 rats. Table 1 shown all group allocation and animal numbers.

Fig. S2 Culture and identification of UCMSCs and HUVECs. (A-B) Morphology of UCMSC under phase contrast microscopy. (C) Identification of UCMSC by fluorescent labeling of CD90 and CD105. (D-E) Morphology of HUVECs under phase contrast microscopy. (F) Identification of HUVEC by fluorescent labeling of vWF and CD31. Scale bars in A represent 250 μm, in B represent 100 μm, in D represent 100 μm, in E represent 200 μm, in F represent 100 μm, in G represent 50 μm.
